# Genetically predicted 486 blood metabolites in relation to risk of esophageal cancer: a Mendelian randomization study

**DOI:** 10.3389/fmolb.2024.1391419

**Published:** 2024-10-02

**Authors:** Caiyan Jia, Dan Yi, Mingze Ma, Qian Xu, Yan Ou, Fanming Kong, Yingjie Jia

**Affiliations:** ^1^ Department of Oncology, First Teaching Hospital of Tianjin University of Traditional Chinese Medicine, Tianjin, China; ^2^ National Clinical Research Center for Chinese Medicine Acupuncture and Moxibustion, Tianjin, China

**Keywords:** Mendelian randomization, blood metabolites, colocalization analysis, SNPs, esophageal cancer

## Abstract

**Background and Objective:**

Enhancing therapy choices for varying stages of esophageal cancer and improving patient survival depend on timely and precise diagnosis. Blood metabolites may play a role in either causing or preventing esophageal cancer, but further research is needed to determine whether blood metabolites constitute a genetic risk factor for the disease. In order to tackle these problems, we evaluated the causal association between esophageal cancer and 486 blood metabolites that functioned as genetic proxies using a two-sample Mendelian randomization (MR) study.

**Methods:**

We utilized two-sample MR analyses to evaluate the causal links between blood metabolites and esophageal cancer. For the exposure, we used a genome-wide association study (GWAS) of 486 metabolites, and a GWAS study on esophageal cancer from Sakaue et al. was used for preliminary analyses. Causal analyses employed randomized inverse variance weighted (IVW) as the main method, supplemented by MR-Egger and weighted median (WM) analyses. Sensitivity analyses included the MR-Egger intercept test, Cochran Q test, MR-PRESSO, and leave-one-out analysis. Additionally, independent esophageal cancer GWAS data were utilized for replication and meta-analysis. FDR correction was applied to discern features with causal relationships. For conclusive metabolite identification, we conducted Steiger tests, linkage disequilibrium score regression, and colocalization analyses. Moreover, we utilized the program MetaboAnalyst 5.0 to analyze metabolic pathways.

**Results:**

This study found an important association between esophageal cancer and three metabolites: 1-linoleoylglycerophosphoethanolamine* [odds ratio (OR) = 3.21, 95% confidence interval (CI): 1.42–7.26, *p* < 0.01], pyroglutamine* (OR = 1.92, 95% CI: 1.17–3.17, *p* < 0.01), and laurate (12:0) (OR = 3.06, 95% CI: 1.38–6.78, *p* < 0.01).

**Conclusion:**

This study establishes a causal link between three defined blood metabolites and esophageal cancer, offering fresh insights into its pathogenesis.

## 1 Introduction

Esophageal cancer seventh in global cancer incidence and is the sixth leading cause of cancer-related deaths (Global Burden of Disease, 2019 Cancer Collaboration et al., 2022). Esophageal cancer develops insidiously, with early symptoms often unnoticed by patients. Diagnosis commonly occurs at an advanced stage, resulting in a poor prognosis (Global Burden of Disease, 2019 Cancer Collaboration et al., 2022). Globally, the incidence of esophageal cancer exhibits distinct geopolitical patterns. Typically, esophageal adenocarcinoma is more prevalent in Western countries, whereas esophageal squamous cell carcinoma (ESCC) is notably more common in Asia’s esophageal cancer belt, as well as in certain areas of East Africa and South America (Shaheen and Ransohoff, 2002). In 2020, an estimated 6,041,000 new cases and 5,440,762 deaths due to esophageal cancer were reported globally (Sung et al., 2021). The heterogeneity and complexity of esophageal cancer lead to most patients being diagnosed at an advanced stage, precluding the possibility of surgery and optimal treatment. Consequently, patients with esophageal cancer have a worse prognosis, increased mortality, and a worse quality of life, evidenced by 5-year survival rates under 20% (Zhang et al., 2023), thereby intensifying the global disease burden.

The incorporation of metabolomics with systems biology has recently offered a new approach in researching disease processes. It remains unclear whether metabolic dysregulation is a cause or a result of esophageal cancer progression. However, substantial evidence indicates that it plays a critical role in both the development and progression of the disease (Wang et al., 2013), beyond changes in glucose metabolism, dysregulation in amino acid, lipid, and protein metabolism has been observed *in vitro* and *in vivo*, as exemplified by the well-known Warburg effect (Gu et al., 2016; Kim et al., 2010; Matsunaga et al., 2018). Although Huang et al. (2020) described 45 blood metabolites associated with esophageal cancer, no thorough investigation has systematically examined the causal connection between esophageal cancer and blood metabolites. The possible causal relationship between alterations in blood metabolite levels and the risk of esophageal cancer requires more investigation. This will aid in understanding the causes of esophageal cancer and enhance its precise prevention and control.

The Mendelian randomization (MR) study is a well-established genetic epidemiological methodology that employs genetic variants as proxies for target exposures to assess the causal relationship between exposures and disease outcomes (Zuccolo and Holmes, 2017). This approach has distinct advantages over traditional observational studies. First, by utilizing random independent classification of DNA during meiosis of alleles, MR analysis avoids traditional confounding. Second, reverse causal effects bias does not occur due to the immutability of the genomes of the propagating germline (Burgess et al., 2015). Third, in most cases, genetic variation is usually accurately measured and reported and is less susceptible to bias and measurement error. Thus, it is particularly useful in assessing risk factors for long-term effects (Hu et al., 2019). In this context, we performed MR analysis with genome-wide association study (GWAS) pooled data to comprehensively explore the causal effects of 486 blood metabolites on esophageal cancer. Additionally, colocalization and metabolic pathway analyses were conducted to explore the gene and protein-level biological processes underlying esophageal cancer. The objective of this study is to uncover the metabolic etiology of esophageal cancer and offer insights into its biological mechanisms.

## 2 Methods

### 2.1 Study design

**FIGURE 1:**
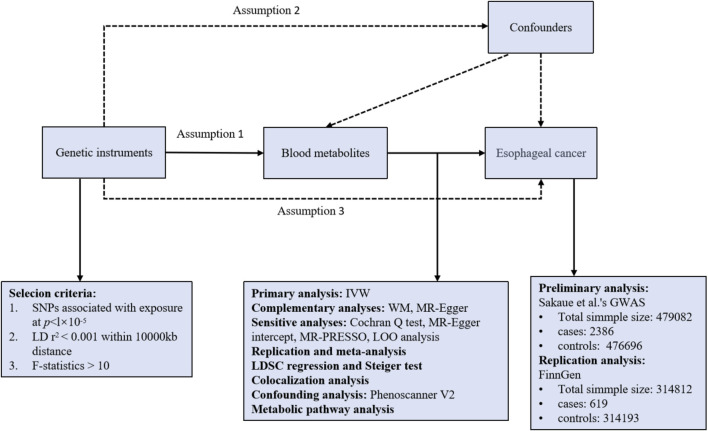
Overview of the study design. Assumption 1, genetic instruments are strongly associated with the exposures of interest; Assumption 2, genetic instruments are independent of confounding factors; Assumption 3, genetic instruments are not associated with outcome and affect outcome only via exposures. LD, linkage disequilibrium; SNPs, single nucleotide polymorphisms; IVW, inverse variance weighted; WM, weighted median; LOO analysis, leave-one-out analysis; MR-PRESSO, MR-Pleiotropy RESidual sum and outlier.

Three presumptions must be met by a valid MR study: (1) IVs are highly correlated with the relevant exposures; (2) IVs are unaffected by confounding variables; (3) IVs have no correlation to outcomes and only have an impact on outcomes through exposures (Boef et al., 2015). All of the MR analyses in this work were performed using R software (version 4.3.2) Two Sample MR and MRPRESSO packages. An overview of the study is shown in Figure 1.

### 2.2 GWAS summary statistics

All analyses of MR in this study were based on summary statistics from the GWAS study of blood metabolites and Esophageal cancer. The summary data for Esophageal cancer is obtained from Sakaue et al. (2021)’s trans-ethnic GWAS meta-analysis, which includes 2,386 cases (998 cases of European origin and 1,388 cases of East Asian origin) and 476,696 controls (475,308 controls of European origin and 159,201 controls of East Asian origin). From the Metabolomics GWAS Server (https://metabolomics.helmholtz-muenchen.de/gwas/), genetic information on blood metabolites was collected. This is the largest analysis on blood metabolite genetic loci to date, with approximately 2.1 million single nucleotide polymorphisms (SNPs) for 486 metabolites associated with genetic variations in humans. Shin et al. (2014)’s high-throughput metabolic profiling and genome-wide association searches were used to accomplish this. Supplementary Table S1 lists the 486 metabolites, with those labeled ‘X-’ indicating unknown chemical properties. The study encompassed 7,824 European participants, comprising 1,768 from Germany’s KORA F4 study and 6,056 from the UK Twin Study. 107 of the 486 metabolites had their chemical characteristics so poorly characterized that they were categorized as unknown. The Kyoto Encyclopedia of Genes and Genomes (KEGG) database also shows that 309 metabolites were chemically confirmed and classified into eight metabolic groups: lipid, nucleotide, energy, peptide, xenobiotic metabolism, carbohydrate, cofactors and vitamins, and amino acid (Kanehisa et al., 2012). As this study was based on publicly available data, no additional ethical approval or consent was required.

### 2.3 IVs selection

MR Research has three core instrumental variable assumptions. (1) Selection of genetic variants as instrumental variables (IVs) associated with exposure factors blood metabolites. To find SNPs associated with exposure factors and to ensure the veracity and accuracy of conclusions about the causal link between blood metabolites and Esophageal cancer risk, the following steps were used to select the best SNPs. After consulting the pertinent literature, the threshold was first modified (*p* < 1 × 10^−5^) due to the small number of SNPs related with metabolites in order to achieve comprehensive and trustworthy results. Then, we eliminated linkage disequilibrium (LD, *R* ^*2*^ < 0.001, kb = 10,000) to clumped SNPs (Yang et al., 2020; Choi et al., 2019). The F-statistic was used to assess the correlation between SNPs and exposure statistical strength, the F-statistic is given by: *F* = $\frac{R^{2}}{1-R^{2}}\;\cdot \frac{N-K-1}{K}$ , where *K* is the amount of SNPs (instrumental variants); *N* is the exposure sample size; *R* ^*2*^ is the instrumental variable (determinant coefficient of regression equation) that describes the extent of exposure, which was calculated as *R* ^*2*^ = 2·(1-EAF)·EAF·*β* ^*2*^, where the genetic variant of interest’s standard error is denoted by *β*, while its effect allele frequency is represented by EAF (Palmer et al., 2012). SNPs with F < 10 were eliminated because they were deemed to have weak genetic variations (Burgess et al., 2013). Subsequently, we isolated SNPs linked to metabolites from the result and eliminated SNPs that were connected to the result (*p* < 1 × 10^−5^). We eliminated allelic inconsistent SNPs (such as A/G vs. A/C) and palindromic effects after further harmonizing SNPs for exposure and outcome. The final data were then submitted to MR analysis (Supplementary Table S2).

### 2.4 Statistical analysis and sensitivity analysis

The primary analysis utilized the random-effects inverse variance weighted (IVW) method to establish a significant causal link between serum metabolites and esophageal cancer (*p* < 0.05). When IVs meet all three main hypotheses, the IVW method yields the most accurate and unbiased estimates, making it the most effective method for MR (Pierce and Burgess, 2013). However, horizontal pleiotropy arises when IVs impact traits beyond the exposure pathway, directly affecting the outcomes and potentially leading to imprecise causal estimates (Thomas and Conti, 2004). MR-Egger regression provides consistent estimates in the presence of genetically pleiotropic IVs (Bowden et al., 2015). The WM method’s strength lies in its ability to yield consistent causal estimates even with over 50% null IVs (Bowden et al., 2016). In repeated comparisons, we used the Benjamini–Hochberg technique to control the false discovery rate (FDR) (Benjamini and Hochberg, 1995). The Benjamini-Hochberg-corrected *p* values (FDR *p*-values) were used as the threshold of significance. Associations that passed the FDR controlled significance levels (FDR *p*-values < 0.05) were considered to be strong evidence of associations, whereas results with *p* less than 0.05 but that failed to pass FDR correction were regarded as suggestive associations.

Sensitivity analysis is essential for detecting horizontal pleiotropy and heterogeneity in the data. Cochran’s Q statistic was employed to assess heterogeneity (Cohen et al., 2015). The MR-Pleiotropy RESidual Sum and Outlier (MR-PRESSO) method was used to identify outliers, which were then excluded for re-analysis if detected (Verbanck et al., 2018). Horizontal pleiotropy was assessed using MR-Egger, a statistically significant difference in intercept terms indicated notable horizontal pleiotropy in the study (Burgess and Thompson, 2017). Robustness of the MR findings was evaluated using used leave-one-out (LOO) analysis. By eliminating each SNP individually, after which MR analysis is conducted to see whether a single SNP significantly affected the outcome.

In conclusion, multiple criteria were used in our thorough screening for blood metabolites that may be causally linked to esophageal cancer: (1) Significant *p*-values (IVW-derived *p* < 0.05, FDR *p* < 0.05); (2) uniformity in magnitude and direction among the 3 MR methods; (3) No horizontal pleiotropy or heterogeneity in the MR results; (4) Lack of significant confounding in MR estimates by individual SNPs.

### 2.5 Replication and meta-analysis

To thoroughly evaluate the robustness of the identified candidate metabolites, we utilized data from the FinnGen consortium (https://www.finngen.fi/fi) for additional validation of the exposure results and their relation to esophageal cancer. FinnGen’s release R10 includes data on 169 esophageal cancer cases and 314,193 other cases. The causal association of blood metabolites with esophageal cancer was confirmed through meta-analysis of 2 MR studies. Using R software (4.3.2), the random effects IVW model was used to implement meta-analysis.

### 2.6 Confounding analysis

While we evaluated the horizontal pleiotropy of MR results through sensitivity analyses to identify SNPs violating MR assumptions, a small residual amount of confounding SNPs may remain. IVs were examined using the Phenoscanner V2 website (http://www.phenoscanner.medschl.cam.ac.uk/) (Staley et al., 2016). Each SNP was evaluated for its association with known esophageal cancer risk factors, including smoking (Doll et al., 1994; Freedman et al., 2007), alcohol consumption (Yokoyama et al., 2002; Prabhu et al., 2014), and obesity (Veugelers et al., 2006; Lagergren, 2011). If any SNPs were associated with these confounders (*p* < 1 × 10^−5^), we reran the MR analysis after their removal to confirm the results’ reliability.

### 2.7 Evaluation of directionality

The Steiger test was used to confirm if the observed causality was influenced by reverse causality. This test examined whether the SNPs included in the study explained more of the esophageal cancer than the metabolites identified (Hemani et al., 2017). When SNP combinations were shown to have no genetic risk for esophageal cancer when compared to metabolites, the results showed that causal inference was not biased (Steiger *p* > 0.05).

### 2.8 Colocalization analysis

After MR analyses identified causality of blood metabolites on esophageal cancer risk, we used co-localization analyses as a sensitivity analysis to s to evaluate whether blood metabolites and esophageal cancer risk share the same causal genetic variant at the GHRL locus (Giambartolomei et al., 2014). Such an analysis can indicate whether the phenotypes are influenced by different causal genetic variants that are in LD, indicative of horizontal pleiotropy and violation of the exclusion restriction assumption (Wallace, 2013). PP.H4 > 75% was considered supportive of colocalization of the two phenotypes.

### 2.9 Metabolic pathway analysis

Using MetaboAnalyst 5.0 (https://www.metaboanalyst.ca/), we performed a metabolic pathway analysis (Jewison et al., 2014) to elucidate the biological mechanisms through which blood metabolites causally affect esophageal cancer, thereby exploring its potential pathogenesis.

## 3 Results

### 3.1 Preliminary analysis

**FIGURE 2:**
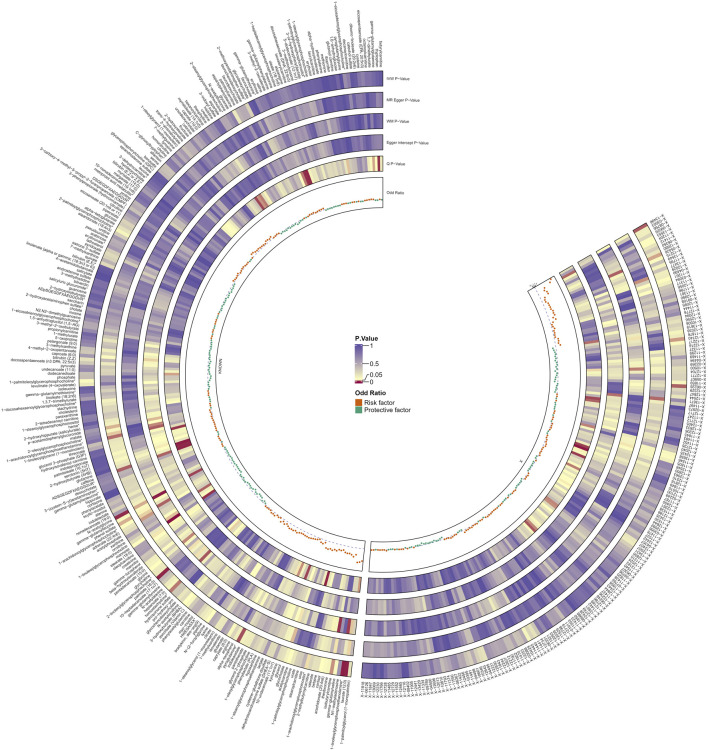
Preliminary MR estimates of the association between blood metabolites and esophageal cancer risk. The shades of color depict the magnitude of the *p-*value.

**FIGURE 3:**
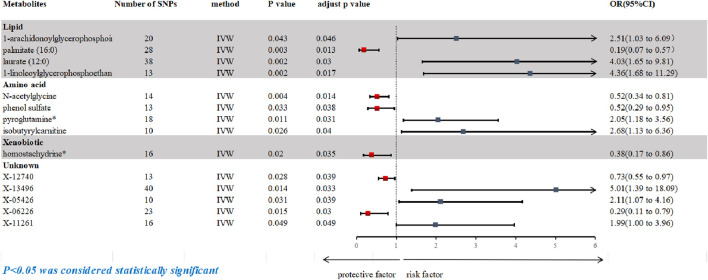
Forest plot based on inverse variance weighted (IVW) analysis was created to show the relationship between blood metabolites and esophageal cancer. The graph shows the correlation between a number of blood metabolites and the risk of esophageal cancer. Each horizontal line represents the OR and 95% CI for how each blood metabolite affects the risk of esophageal cancer. IVW, inverse variance weighted; CI, confidence interval; SNPs, single nucleotide polymorphisms; OR, odds ratio.

**FIGURE 4:**
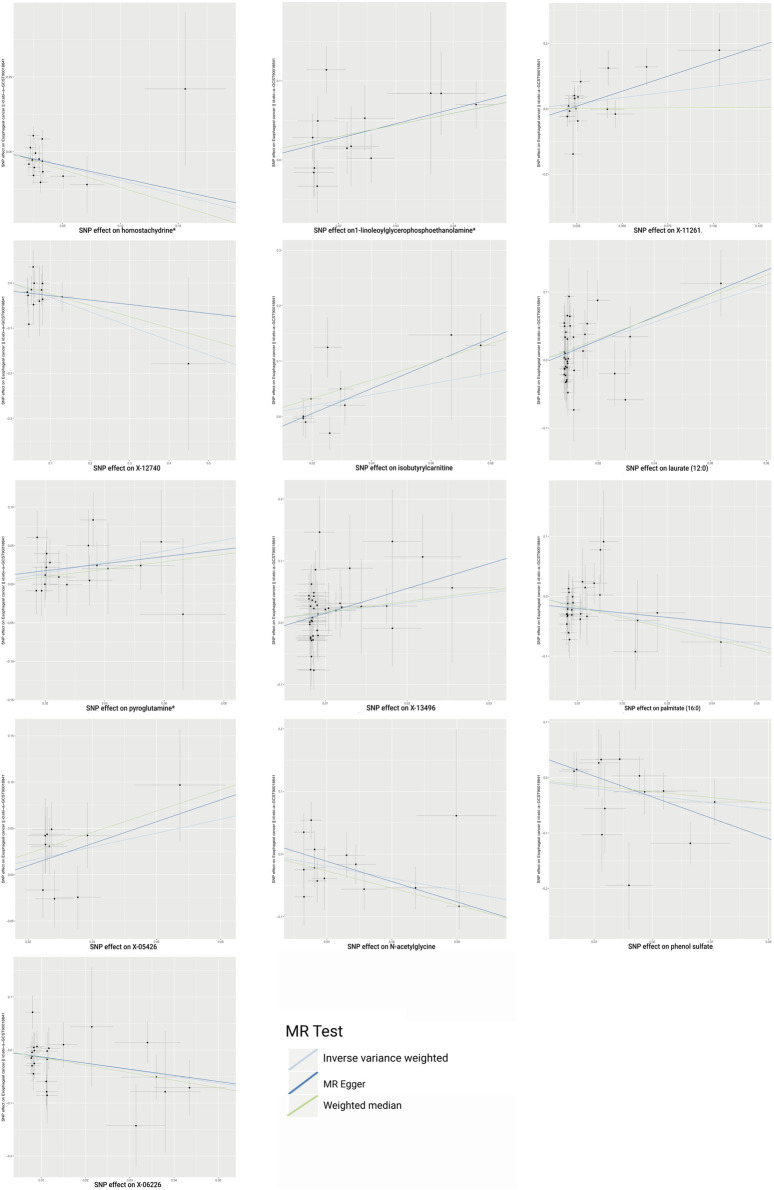
Scatterplot shows estimates that are strongly linked (IVW determined *p* < 0.05) and directionally consistent. SNP, single nucleotide polymorphisms.

Following rigorous quality control of the IVs, our MR study identified 486 blood metabolites. Preliminary research depicted in Figure 2 points to a relationship between blood metabolites and the risk of esophageal cancer. The refined IVs comprised 276 SNPs. IVW analysis identified 14 metabolites potentially causally related to esophageal cancer, consisting of 9 metabolites with known chemical properties and 5 with unknown properties, as shown in Figure 3. Figure 3 illustrates that the nine identified metabolites are chemically classified into amino acids, lipids, and xenobiotics. After FDR, no metabolites were found that still had a significant effect. Palmitate (16:0) (odds ratio [OR] = = 0.19, 95% confidence interval [CI]: 0.07–0.57, *p* = 0.003, adjust *p* = 0.013), X-06226 (OR = 0.29, 95%CI: 0.11–0.79, *p* = 0.015, adjust *p* = 0.03); phenol sulfate (OR = 0.52, 95%CI: 0.29–0.95, *p* = 0.033, adjust *p =* 0.038); N-acetylglycine (OR = 0.52, 95%CI: 0.34–0.81, *p =* 0.004, adjust *p =* 0.014); homostachydrine^*^ (OR = 0.38, 95%CI: 0.17–0.86, *p =* 0.020, adjust *p =* 0.035); X-12740 (OR = 0.73, 95%C: 0.55–0.9, *p =* 0.028, adjust *p =* 0.039) was negatively associated with the risk of esophageal cancer, whereas laurate (12:0) (OR = 4.03, 95%CI: 1.65–9.81, *p =* 0.002, adjust *p =* 0.03); X-05426 (OR = 2.11, 95%CI: 1.07–4.16, *p =* 0.031, adjust *p =* 0.039); X-11261 (OR = 1.99, 95%CI: 1.00–3.96 *p =* 0.049, adjust *p =* 0.049); 1-linoleoylglycerophosphoethanolamine^*^9 (OR = 4.36, 95%CI: 1.68–11.29, *p =* 0.002, adjust *p =* 0.017); pyroglutamine^*^ (OR = 2.05, 95%CI: 1.18–3.56, *p =* 0.011, adjust *p =* 0.031); isobutyrylcarnitine (OR = 2.68, 95%CI: 1.13–6.36, *p =* 0.026, adjust *p =* 0.04); X-1349(OR = 5.01, 95%CI: 1.39–18.09, *p =* 0.014, adjust *p =* 0.033); 1-arachidonoylglycerophosphoinositol^*^ (OR = 4.36, 95%CI: 1.68–11.29, *p =* 0.043, adjust *p =* 0.046) were positively correlated with the risk of esophageal cancer. For 1-arachidonoylglycerophosphoinositol^*^ (OR = 4.36, 95%CI: 1.68–11.29, *p* = 0.043, *β* = 0.919), the MR-Egger method showed different results (OR = 0.743, 95%CI: 0.108–5.129, *p* = 0.767, *β* = −0.297). Despite no horizontal pleiotropy in MR-Egger regression (*p*_intercept = 0.184), potential outliers may cause inconsistencies between MR-Egger and IVW results. Thus, further investigations are needed to validate its association with esophageal cancer. In summary, IVW-derived estimates showed significance (*p* < 0.05), and the direction and magnitude of the IVW, MR-Egger, and WM estimates aligned consistently, as depicted in Figure 4. The Cochran Q test indicated no heterogeneity among the selected SNPs, as evidenced by Q_pval values for IVW and MR-Egger all exceeding 0.05. Homostachydrine^*^ was excluded by the MR-PRESSO method and the rest were not detected with outliers (*p* > 0.05). MR-PRESSO results did not indicate heterogeneous SNPs post-outlier removal (Supplementary Table S3). Cochran Q test and MR-Egger cutoff test results (both *p* > 0.05) strongly suggest the lack of pleiotropy and heterogeneity (Table 1). The results of the LOO analysis indicated that no single SNP introduced bias into the MR estimates (Supplementary Figure S1). Twelve blood metabolites were identified as subjects for more investigation.

**TABLE 1:** Supplementary and sensitivity analysis examine the relationship between blood metabolites and esophageal cancer.

Metabolites	N	MR analysis			Heterogeneity		Pleiotropy	
		Methods	*p*	OR (90%CI)	Q	Q_*p*	Intercept	*p*
Lipid								
1-arachidonoylglycerophosphoinosito1^*^	20	MR Egger	0.77	0.74 (0.11–5.13)	22.19	0.22	0.028	0.18
	20	Weighted median	0.95	1.04 (0.32–3.39)				
palmitate (16:0)	28	MR Egger	0.50	0.48 (0.06–4.05)	19.49	0.81	−0.013	0.35
	28	Weighted median	0.05	0.17 (0.03–1.00)				
laurate (12:0)	38	MR Egger	0.04	5.56 (1.14–27.09)	42.15	0.22	−0.005	0.63
	38	Weighted median	0.04	4.76 (1.07–21.09)				
1-linoleoylglycerophosphoethanolamine^*^	13	MR Egger	0.19	6.50 (0.47–89.15)	16.70	0.12	−0.010	0.75
	13	Weighted median	0.01	4.39 (1.39–13.93)				
Amino acid								
N-acetylglycine	14	MR Egger	0.03	0.34 (0.14–0.81)	11.85	0.46	0.021	0.28
	14	Weighted median	0.00	0.40 (0.23–0.72)				
phenol sulfate	13	MR Egger	0.05	0.15 (0.03–0.80)	13.23	0.28	0.059	0.15
	13	Weighted median	0.17	0.60 (0.29–1.25)				
pyroglutamine^*^	18	MR Egger	0.55	1.59 (0.37–6.84)	8.32	0.94	0.008	0.72
	18	Weighted median	0.24	1.62 (0.73–3.62)				
isobutyrylcamitine	10	MR Egger	0.04	9.60 (1–50–61.50)	7.61	0.47	−0.040	0.17
	10	Weighted median	0.00	5.18 (1.74–15.41)				
Xenobiotic								
homostachydrine^*^	16	MR Egger	0.57	0.43 (0.03–7.08)	25.82	0.03	−0.004	0.93
	16	Weighted median	0.01	0.30 (0.13–0.74)				
Unknown								
X-05426	10	MR Egger	0.42	3.26 (0.22–48.75)	8.67	0.37	−0.014	0.75
	10	Weighted median	0.02	3.14 (1.20–8.21)				
X-06226	23	MR Egger	0.21	0.32 (0.06–1.79)	21.14	0.45	−0.002	0.90
	23	Weighted median	0.06	0.24 (0.05–1.07)				
X-11261	16	MR Egger	0.06	6.24 (1.09–35.85)	17.43	0.23	−0.037	0.19
	16	Weighted median	0.93	1.04 (0.42–2.56)				

### 3.2 Replication and meta-analysis

**FIGURE 5:**
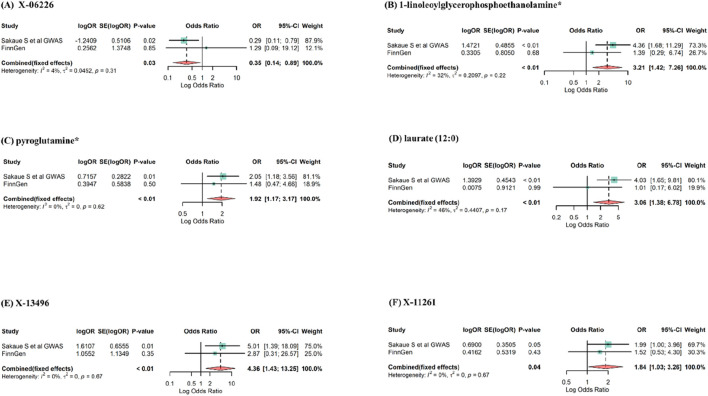
Meta-analysis of significantly associated (IVW derived *p* < 0.05) between blood metabolites and esophageal cancer. OR, odds ratio; 95% CI, 95%confidence interval. **(A)** X-06226, **(B)** 1-linoleoylglycerophosphoethanolamine^*^, **(C)** pyroglutamine^*^, **(D)** laurate (12:0), **(E)** X-13496, **(F)** X-11261.

To bolster the credibility of our findings, we replicated the MR analysis using a separate GWAS dataset for esophageal cancer. The other GWAS dataset revealed a consistent trend for the candidate metabolites, albeit the results were not statistically significant, most likely due to major sample size differences. The meta-analysis identified 6 blood metabolites (3 known and 3 unknown) with potential effects on esophageal cancer, as shown in Figure 5. Higher levels of X-06226 (OR = 0.35, 95%CI: 0.14–0.89, *p* = 0.03) were associated with reduced esophageal cancer risk. Conversely, elevated levels of 1-linoleoylglycerophosphoethanolamine^*^ (OR = 3.21, 95%CI: 1.42–7.26, *p* < 0.01), pyroglutamine^*^ (OR = 1.92, 95%CI: 1.17–3.17, *p* < 0.01), laurate (12:0) (OR = 3.06, 95%CI: 1.38–6.78, *p* < 0.01), X-13496 (OR = 4.36, 95%CI: 1.43–13.25, *p* < 0.01), and X-11261 (OR = 1.84, 95%CI: 1.03–3.26, *p* = 0.04) appeared to increase the risk. Although palmitate (16:0), isobutyrylcarnitine, N-acetylglycine, phenol sulfate, X-05426, and X-12740 consistently aligned in both MR analyses, they were excluded due to non-significant estimates in the meta-analysis. Although the six metabolites mentioned above were not significant in the replication analysis, their consistent directionality suggests more than mere coincidence. This pattern in MR estimation might be due to differences in sample size. Therefore, by expanding the sample size and statistical robustness through meta-analysis, we identified metabolites causally linked to esophageal cancer.

### 3.3 Confounding analysis

While sensitivity analysis excluded SNPs violating our estimates, to meet assumption 2 (IVs are independent of confounders), we checked in Phenoscanner if all SNPs linked to the six metabolites were unaffected by risk factors for esophageal cancer, including alcohol consumption, smoking, and obesity. Our analysis revealed no confounders for X-06226, pyroglutamine^*^, and X-1349. However, among other IVs related to three metabolites, five SNPs were associated with confounders (Supplementary Table S4). After excluding these SNPs and reapplying MR analysis, we determined that laurate (12:0) (OR = 4.02, 95%CI: 1.62–9.96, *p* = 0.003) and 1-linoleoylglycerophosphoethanolamine^*^ (OR = 4.22, 95%CI: 1.73–10.34, *p* = 0.002) maintained a stable association with esophageal cancer. In contrast, X-11261 (OR = 1.67, 95%CI: 0.55–2.48, *p* = 0.690) showed an unstable association.

### 3.4 Evaluation of directionality

Furthermore, the Steger test was conducted to investigate the possibility of an inverse causal relationship between the three chemically identified metabolites and esophageal cancer. The Steger test results did not indicate a reverse causal effect between these metabolites and esophageal cancer (*p* < 0.05) (Supplementary Table S5).

### 3.5 Colocalization analysis

Colocalization analysis results revealed a low probability of shared genetic variation between the metabolites and esophageal cancer (laurate (12:0): PPH4 = 22%; pyroglutamine^*^: PPH4 = 3%; 1-linoleoylglycerophosphoethanolamine^*^: PPH4 = 21%) (Supplementary Table S6). This implies that the MR analysis is not bias by horizontal pleiotropy.

### 3.6 Metabolic pathway analysis

Utilizing three identified metabolites, we pinpointed three metabolic pathways potentially implicated in esophageal cancer pathogenesis (Supplementary Table S7). The pathways of Mitochondrial Beta-Oxidation of Medium Chain Saturated Fatty Acids, Beta Oxidation of Very Long Chain Fatty Acids, and Fatty Acid Biosynthesis are suggested as underlying biological mechanisms in the development of esophageal cancer. Significantly, laurate (12:0) is a component in all these metabolic pathways. This suggests a crucial role for laurate (12:0) and its associated metabolic pathways in the pathogenesis of esophageal cancer.

## 4 Discussion

In the current research, we merged two large-scale GWAS data to examine the causal effects of 486 blood metabolites on esophageal cancer by genetic proxy through a rigorous MR design. We confirmed causal associations between 3 known blood metabolites and esophageal cancer risk. Specifically high levels of 1-linoleoylglycerophosphoethanolamine^*^, pyroglutamine^*^, and laurate (12:0) were genetically predisposed to increase susceptibility to esophageal cancer. Three metabolic pathways that might have a role in the biological mechanisms behind esophageal cancer have been discovered. To the best of what we know, this is the first MR research to use colocalization analysis and metabolic pathways in conjunction with the largest available blood metabolite GWAS data to investigate the causal relationship with esophageal cancer. These results underline the critical role that blood metabolites play in the pathophysiology of esophageal cancer and offer insightful information for potential studies on the detection and avoidance of the disease.

The high morbidity and mortality rates of esophageal cancer have placed a tremendous burden on people globally in recent years, making early detection and earlier treatment of the illness an urgent goal. Esophageal cancer is associated with a variety of dietary habits. A meta-analysis showed that meat, red meat, saturated fat, salt intake, and the temperature of the food were positively associated with the incidence of esophageal cancer (Castro et al., 2018). An important part of preventing esophageal cancer is understanding the dietary exposure factors linked to the disease. In addition, animal, or saturated fats, including butter, have been positively associated with a high incidence of esophageal cancer (Tullio et al., 2020). However, the evidence to date is not sufficient to demonstrate exactly how they promote cancer development, and many studies are still needed to prove it. The perceived value of metabolites in esophageal tissue is being investigated with more attention due to the advent of metabolomics technology. Notably, because blood metabolites concurrently record endogenous and external activities, they visually offer a picture of biological operations (Rappaport et al., 2014). The contribution of metabolites to early screening and prevention of esophageal cancer is restricted because of the uncertain causal link between the two, despite prior research showing that they are advantageous to therapy and have a part in the molecular processes of esophageal cancer. In order to clarify the causative association between blood metabolites and esophageal cancer and to investigate the metabolic pathways involved, we thus carried out a critical MR study. This study served as a guide for future research aimed at fully understanding the pathophysiology of esophageal cancer.

The identification of biomarkers is a significant therapeutic contribution of this work. Our study supports the positive association between laurate (12:0), pyroglutamine^*^, 1-linoleoylglycerophosphoethanolamine^*^ and the risk of esophageal cancer by combining genetics and metabolomics from a causality perspective. And laurate (12:0) is implicated in three significantly enhanced metabolic pathways: Mitochondrial Beta-Oxidation of Medium Chain Saturated Fatty Acids, Beta Oxidation of Very Long Chain Fatty Acids, and Fatty Acid Biosynthesis. Laurate (12:0), also known as dodecanoic acid, is one of the saturated fatty acids and a medium chain fatty acid (MCFA), which is the body’s energy supply fuel (Schönfeld and Wojtczak, 2016). Saturated fatty acids are a class of fatty acids (FA) that do not contain unsaturated double bonds and are one of the basic components that make up lipids (Schönfeld and Wojtczak, 2016). Saturated fatty acids are risk factors for several cancers. Laurate (12:0), one of the saturated fatty acids, can be ingested through the diet or produced through the digestion of medium and long chain fatty acids at the small intestine. These medium-chain fatty acids are absorbed through the intestinal wall and enter the blood circulatory system to reach all parts of the body to perform their physiological roles. Related studies have shown that lauric acid can stimulate mammary cell proliferation in mice by activating the GPR84 and PI3K/Akt signaling pathways (Meng et al., 2017). In *in vitro* studies, lauric acid can activate macrophages by promoting signaling at Toll-like receptors (TLR2 heterodimer and TLR4 homodimer) (Huang et al., 2012; Lee et al., 2004), and it can also play a pro-inflammatory role by activating macrophages via the MCFA receptor GPR84 (Suzuki et al., 2013).

Increased FA biosynthesis is one of the hallmarks of abnormal metabolism in tumor cells. Currie et al. (2013) and Cao (2019) have shown the significance of FAs production for cancer cell proliferation and survival. The increase in fatty acid biosynthesis may be a response to the high metabolic demands of cancer cells or an adaptation to the reduced availability of serum-derived lipids in the tumor microenvironment. Systemic mobilisation of lipids from adipose tissue during cancer cachexia promotes tumor growth (Argilés et al., 2014). Thus, the significant increase in laurate (12:0) in esophageal cancer patients may be an adaptation to the high energy expenditure of cancer patients. FA biosynthesis is considered a potential therapeutic target for cancer. A study showed that saturated free fatty acid palmitate induced EMT in hepatocellular carcinoma (HCC) cells through activation of the Wnt/catenin and TGF-AKT pathways (Nath et al., 2015). Furthermore, in studies related to breast cancer, free fatty acids (FFA) are circulating plasma factors that are associated with increased proliferation and invasiveness of estrogen receptor alpha (ERα)-positive (ER +) breast cancer cells. FFA activates ERα and mTOR pathways in breast cancer cells and alters metabolism (Madak-Erdogan et al., 2019). Therefore, we speculate that the development of esophageal cancer affects fatty acid synthesis in the body, which may contribute to cancer progression through increased uptake of various fatty acids such as lauric acid.

Though its function in esophageal cancer is yet unknown, 1-linoleoyglycerophosphoethanolamine^*^ is substantially expressed in colorectal cancer tissues (Mika et al., 2020) and has been demonstrated in a MR experiment to be protective against colorectal cancer (Yun et al., 2023). Furthermore, 1-linoleoylglycerophosphoethanolamine is a significant component of the phosphatidy-lethanolamine (PE) (van der Veen et al., 2017). PE, a significant constituent of phospholipids in cell membranes, is linked to anxiety and plays a crucial role in preserving the stability of cell structure. According to Reichel’s study (Reichel et al., 2015), people with alcohol dependence frequently felt anxious when they abstained from drinking and had higher plasma PE concentrations when they did not. Furthermore, Yang et al. (2020)’s investigation into the connection between metabolites and certain mental conditions discovered that 1-linoleoylglycerophosphatidylethanolamine was linked to a higher risk of major depressive disorder; this finding was subsequently corroborated by another research (Xiao et al., 2022). This seems to suggest that 1-linoleoylglycerophosphatidylethanolamine may well be able to affect depression, which might therefore act as a mediator in the development of esophageal cancer. So far, there are no reports on Pyroglutamine and esophageal cancer, and there is only one study suggesting that Pyroglutamine may be associated with prostate carcinogenesis, but the mechanism is not clear, and more research is necessary to determine the connection between pyroglutamine and cancer.

Our study has several strengths. This is the first and most important point. This is the most thorough and organized investigation of the causative association between blood metabolites and esophageal cancer, and the first MR research to evaluate the relationship between blood metabolites and esophageal cancer. With this kind of design, the limits of confounders that are frequently present in conventional observational studies are mitigated, and there may be more proof of a causal association between exposure and result. Secondly, our results are compelling. In terms of direction and sensitivity analysis, all 3 MR estimations exhibit good consistency. Third, we conducted replication studies and meta-analyses using extra GWAS data and incorporated GWAS datasets from several populations in our study. As a result, our conclusions are now more trustworthy and thorough. Finally, our study provides new insights into the molecular pathways involved in esophageal carcinogenesis by combining genomics and metabolomics.

The present investigation is not without limits. First, our MR analysis set a little relaxed threshold due to the modest number of SNPs related with metabolites. Nonetheless, F-statistics more than 10 were found for every metabolite-associated SNP, indicating strong IVs efficacy; consistent causal direction support from the Steiger test results provides confidence to our relaxed threshold choice. Secondly, even if the database utilized for this research contained two sizable populations with East Asian and European ancestry, it is still not representative of the entire world’s population. Furthermore, although MR analyses provide valuable insights into etiology, it is important to note that our findings should be validated by rigorous randomized controlled trials and basic research before clinical application.

## 5 Conclusion

In conclusion, this MR study showed that 3 known blood metabolites are causally associated with esophageal cancer. Three metabolic pathways that may be associated with the development of esophageal cancer were also identified. The identification of these serum metabolites offers important new information on the early detection, avoidance, and therapy of esophageal cancer in addition to the planning of upcoming clinical trials, even if more validation of the experimental data is still required. The pathophysiology and etiology of esophageal cancer may also be explored in reference form using the combined genomic and metabolomic MR study.

## Data Availability

The original contributions presented in the study are included in the article/Supplementary Material, further inquiries can be directed to the corresponding authors.
