# Supplementary material for: Genetically predicted 486 blood metabolites in relation to risk of esophageal cancer: a Mendelian randomization study
Source: Front Mol Biosci. 2024 Oct 2;11:1391419. doi: 10.3389/fmolb.2024.1391419 (PMC11479936; doi:10.3389/fmolb.2024.1391419)

**Supplementary Materials**

**Genetically predicted 486 blood metabolites in relation to risk**

**of Esophageal cancer: A Mendelian randomization study**

**Supplementary Figure 1 Forest plots for the Mendelian randomization (MR) leave-one-out analysis of the significant inverse variance weighted (IVW) estimates.**


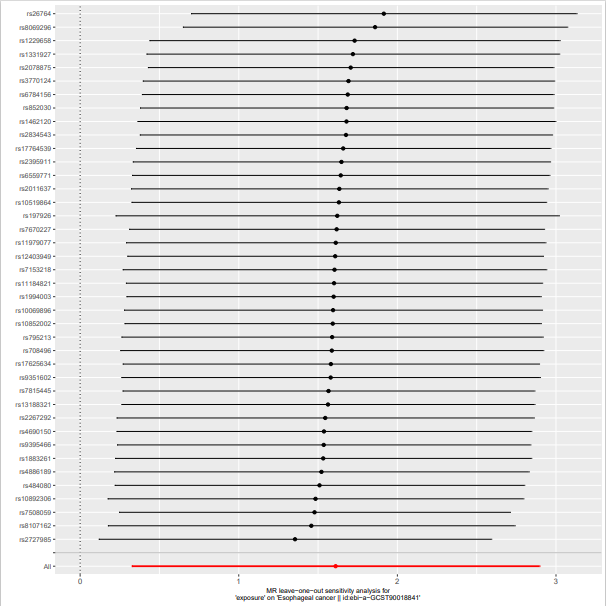

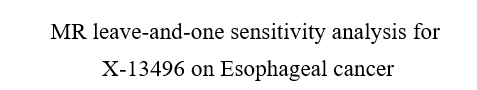

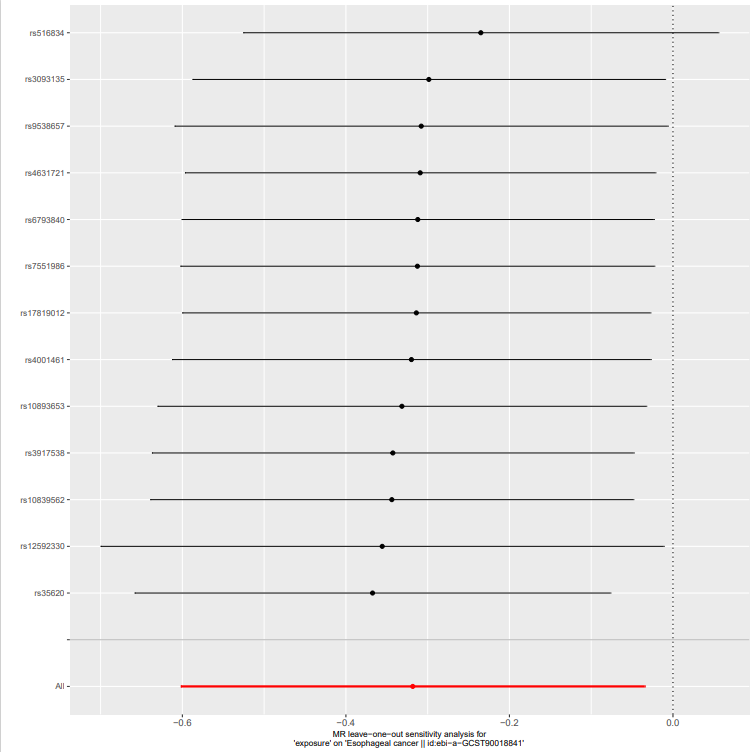

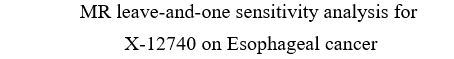

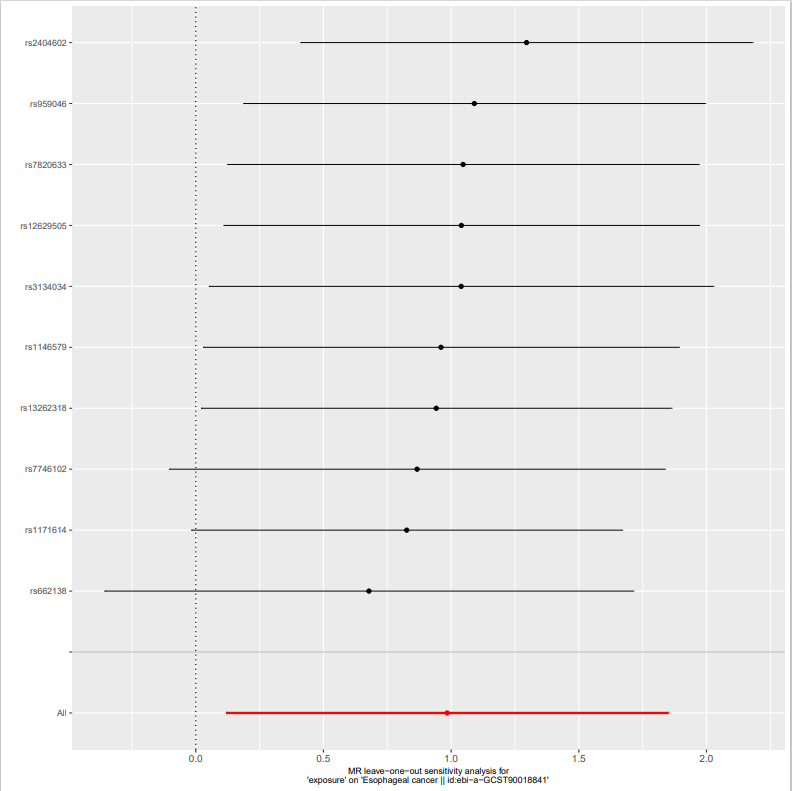

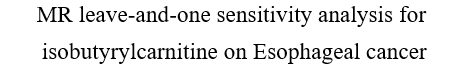

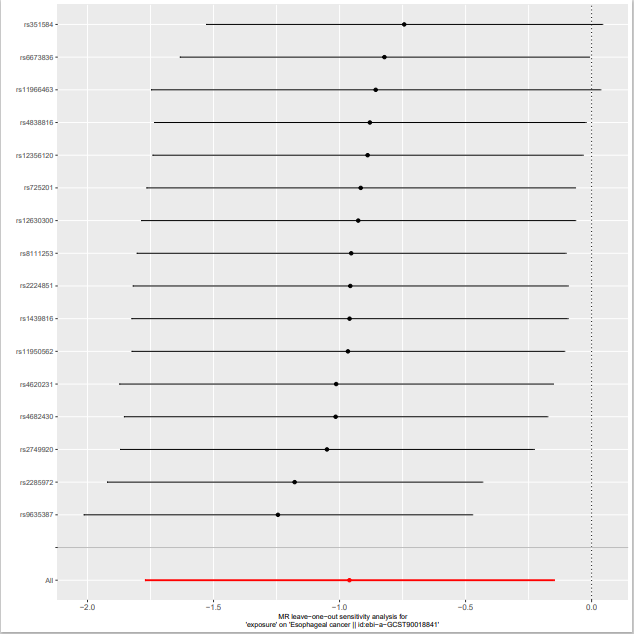

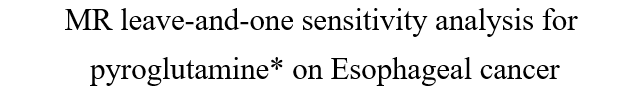

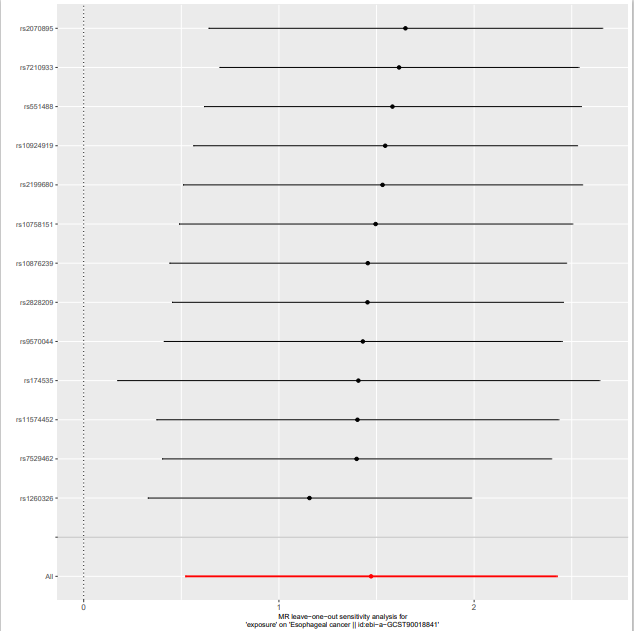

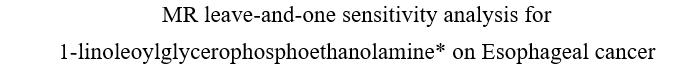

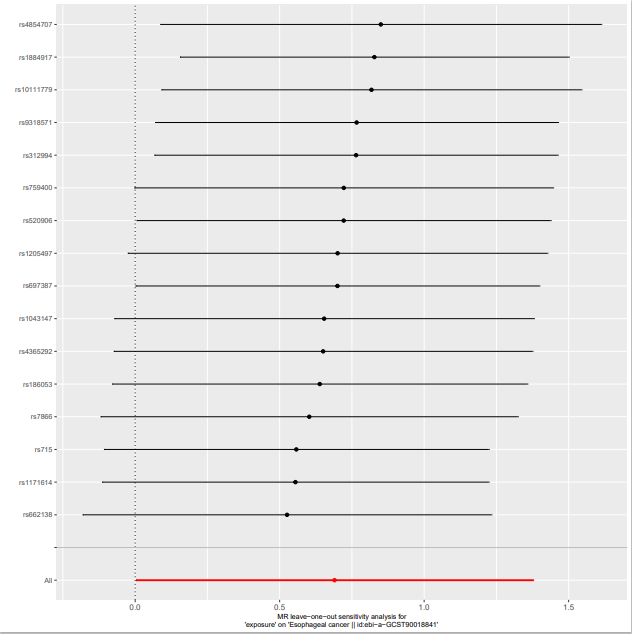

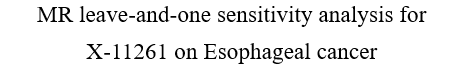

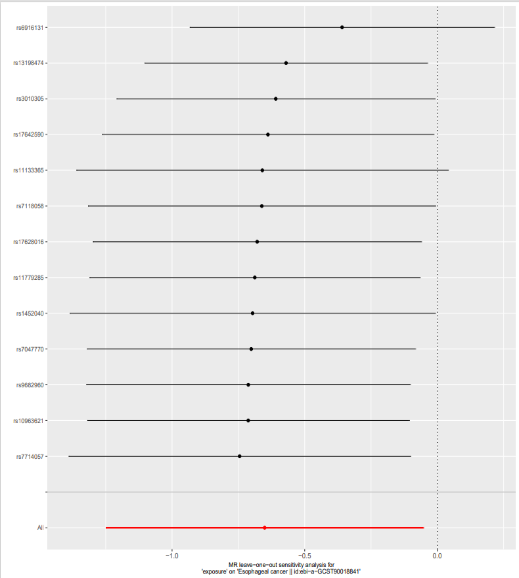

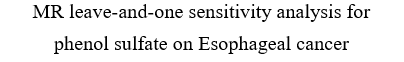

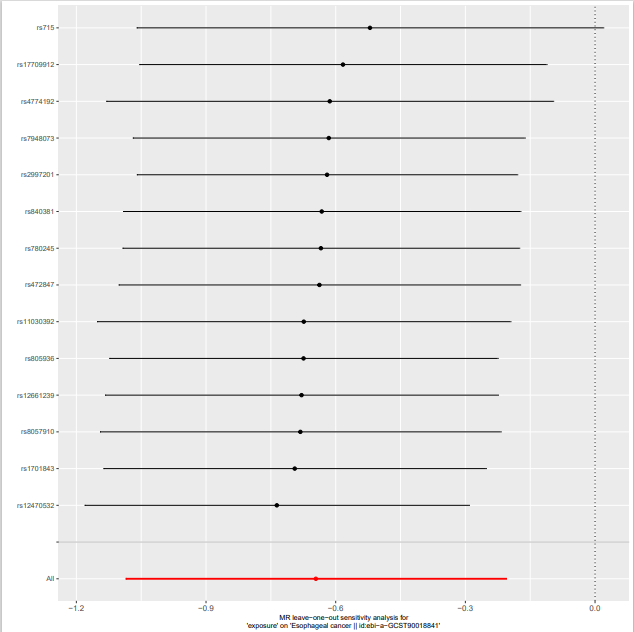

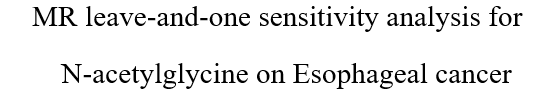

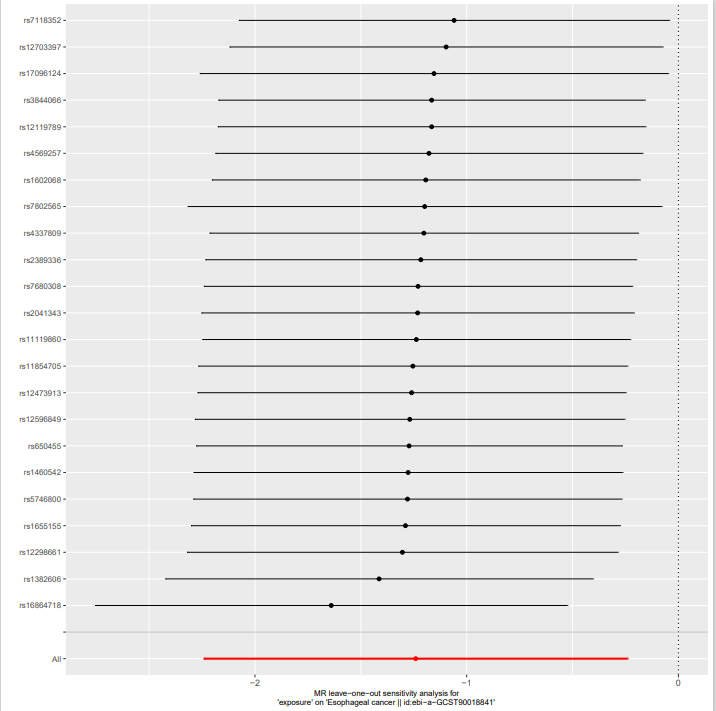

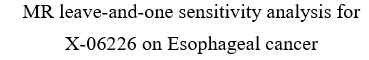

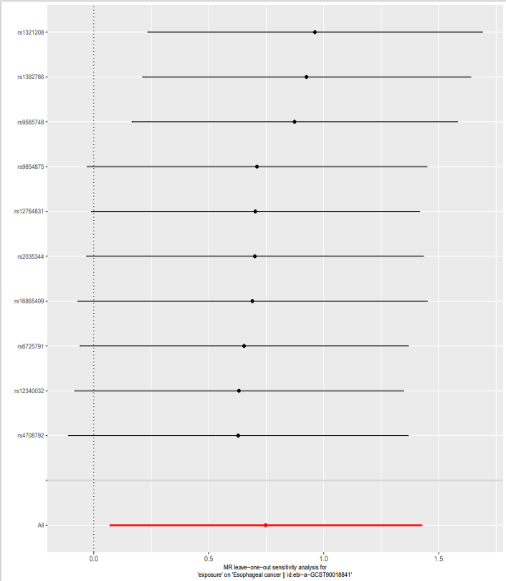

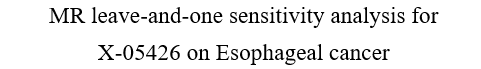

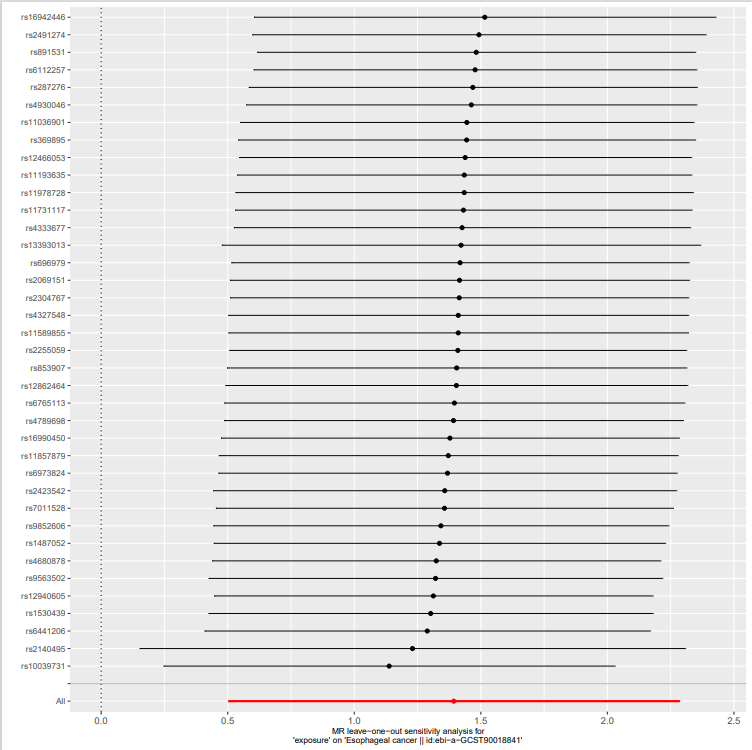

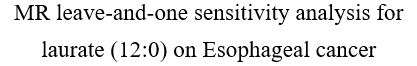

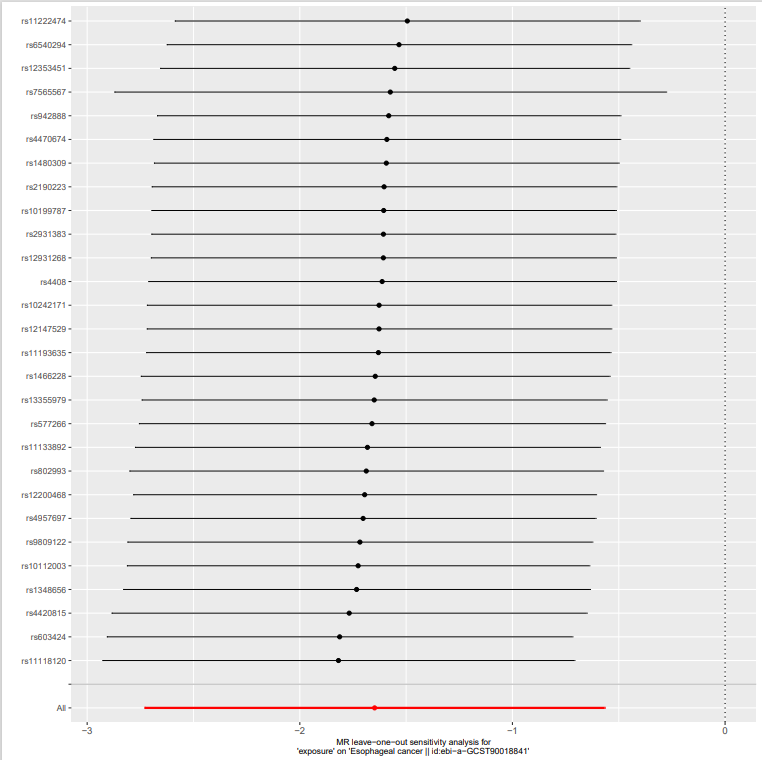

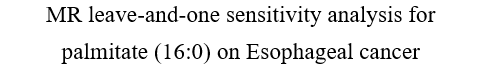

Supplement: Supplementary file 1 [file DataSheet1.DOCX]
